# Calcimimetic R-568 and Its Enantiomer S-568 Increase Nitric Oxide Release in Human Endothelial Cells

**DOI:** 10.1371/journal.pone.0030682

**Published:** 2012-01-25

**Authors:** Mario Bonomini, Annalisa Giardinelli, Caterina Morabito, Sara Di Silvestre, Moreno Di Cesare, Natalia Di Pietro, Vittorio Sirolli, Gloria Formoso, Luigi Amoroso, Maria Addolorata Mariggiò, Assunta Pandolfi

**Affiliations:** 1 Institute of Nephrology-Department of Medicine, University “G. d'Annunzio”, Chieti-Pescara, Italy; 2 Department of Biomedical Sciences, University “G. d'Annunzio”, Chieti-Pescara, Italy; 3 Department of Neuroscience and Imaging, University “G. d'Annunzio”, Chieti-Pescara, Italy; 4 Department of Medicine and Aging Sciences, University “G. d'Annunzio”, Chieti-Pescara, Italy; 5 Aging Research Center, Centro Scienze dell'Invecchiamento (Ce.S.I.), “G. d'Annunzio” University Foundation, Chieti-Pescara, Italy; University of Tor Vergata, Italy

## Abstract

**Background:**

Calcimimetics, such as R-568, are thought to activate G protein-linked Ca^2+^-sensing receptor (CaSR) by allosterically increasing the affinity of the receptor for Ca^2+^ allowing for efficient control of uremic hyperparathyroidism. Several recent studies suggest they possess additional vascular actions. Although it has been postulated that calcimimetics may have a direct effect on CaSR in the blood vessels, further studies are needed to elucidate their vascular CaSR-dependent versus CaSR-independent effects.

**Methodology/Principal Findings:**

Focusing on human umbilical vein endothelial cells (HUVECs), we studied the CaSR expression and distribution by Immunofluorescence and Western Blot analysis. CaSR function was evaluated by measuring the potential effect of calcimimetic R-568 and its enantiomer S-568 upon the modulation of intracellular Ca^2+^ levels (using a single cell approach and FURA-2AM), in the presence or absence of Calhex-231, a negative modulator of CaSR. To address their potential vascular functions, we also evaluated R- and S-568-stimulated enzymatic release of Nitric Oxide (NO) by DAF-2DA, by Nitric Oxide Synthase (NOS) radiometric assay (both in HUVECs and in Human Aortic Endothelial Cells) and by measuring eNOS-ser1177 phosphorylation levels (Immunoblotting). We show that, although the CaSR protein was expressed in HUVECs, it was mainly distributed in cytoplasm while the functional CaSR dimers, usually localized on the plasma membrane, were absent. In addition, regardless of the presence or absence of Calhex-231, both R- and S-568 significantly increased intracellular Ca^2+^ levels by mobilization of Ca^2+^ from intracellular stores, which in turn augmented NO release by a time- and Ca^2+^-dependent increase in eNOS-ser1177 phosphorylation levels.

**Conclusions/Significance:**

Taken together, these data indicate that in human endothelium there is no stereoselectivity in the responses to calcimimetics and that CaSR is probably not involved in the action of R- and S-568. This suggests an additional mechanism in support of the CaSR-independent role of calcimimetics as vasculotrope agents.

## Introduction

Calcimimetics represent a new therapeutic opportunity for treating mineral metabolism disorders related to secondary hyperparathyroidism in patients suffering from chronic kidney disease and uremia [1].

In humans, the parathyroid gland cells can sense small fluctuations in plasma calcium (Ca^2+^) levels by virtue of a cell surface calcium sensing receptor (CaSR), which is a low-affinity G protein-coupled receptor consisting of 1078 amino acid residues [2]–[4]. Although Ca^2+^ itself can be considered the main activator of CaSR, there is a list of known direct (type I agonists) and indirect allosteric (type II agonists) regulators of CaSR functions. Thus, it seems to be a promiscuous receptor that senses changes in multiple physiologic parameters [5]. In the parathyroid glands the class of calcimimetics binds in a stereospecific way [6], [7] to CaSR, and through allosteric activation renders it more sensitive to extracellular Ca^2+^ concentration ([Ca^2+^]o), subsequently resulting in reduction of parathyroid hormone (PTH) secretion and improvement of calcium phosphate products [8].

In addition to the organs specifically involved in Ca^2+^ homeostasis, CaSR is widely expressed in many other tissues including blood vessels [9]. Thus, although most studies on CaSR signaling have been performed in parathyroid cells and human embryonic kidney cells stably transfected with CaSR, evidence of a functional CaSR in endothelial cells from animal model and human blood vessels has been provided [10]–[12].

Recently GPRC6A, a novel G protein-coupled receptor (designated family C, group 6, subtype A) that is sensitive to Ca^2+^ and closely related to CaSR [13] has been identified in endothelium of rat mesenteric and coronary arteries [14] and, notably, can be activated by NPS R-568 [15], a known positive allosteric modulator of the CaSR.

Thus, although the vascular effect of calcimimetics may depend on calcimimetic-induced suppression of parathyroid hypertensive factors [16], direct effects on blood vessels by calcimimetics, via CaSR and/or other mechanisms, cannot be excluded [17].

According to this hypothesis, recent studies have demonstrated in vivo hypotensive effects by calcimimetic agents (type II agonists) in both normotensive [18] and spontaneously hypertensive rats [19]. In addition, Koleganova et al. [20] have now further extended the above observations concerning R-568 on vascular remodeling both in control and in uremic rats.

More recently, it has been demonstrated in vitro that human aortic endothelial cells express a functional CaSR that responds to the endogenous polyamine spermine (CaSR type I agonist) by an increase in intracellular calcium levels([Ca^2+^]i), leading to the production of Nitric Oxide (NO) [11]. NO is a gaseous molecule which has pleiotropic effects in the regulation of vascular tone [21] and is able to maintain vascular homeostasis [22].

Of note, Nakagawa and coll. [23] have shown acute cardiovascular effects in rats by the type II agonist calcimimetic R-568, and its enantiomer S-568. Since this molecule has no or very little activity on the CaSR, the hypotensive effect of R-568 was most likely not mediated via CaSR.

Furthermore, ex vivo studies in isolated arteries have demonstrated some CaSR-independent relaxant effects by calcimimetics, predominantly acting by inhibiting Ca^2+^ influx through L-type Ca^2+^ channels into vascular smooth muscle [24]. Thus, although multiple lines of evidence suggest that calcimimetics might participate in the modulation of a number of vascular functions, we still need to elucidate the CaSR-dependent versus CaSR-independent effects of calcimimetics.

Since it has previously been demonstrated that human aortic endothelial cells express a functional CaSR [11], the principal aims of the present study were first to assess the presence and localization of CaSR in human vein endothelium and then to evaluate its potential function by measuring the effect of calcimimetics R-568 and its enantiomer S-568 on the modulation of intracellular Ca^2+^ levels. Secondly, in view of the key role of intracellular calcium levels in the modulation of endothelial Nitric Oxide Synthase (eNOS) enzymatic activity [25] and the fundamental actions of released NO in the modulation of vascular functions, we evaluated the effects of calcimimetics R-568 and S-568 on the mechanisms of eNOS activation and NO production in both human venous and aortic endothelial cells (HAECs).

## Results

### Immunofluorescence and Western blot analysis for CaSR expression in HUVECs

**Figure 1:**
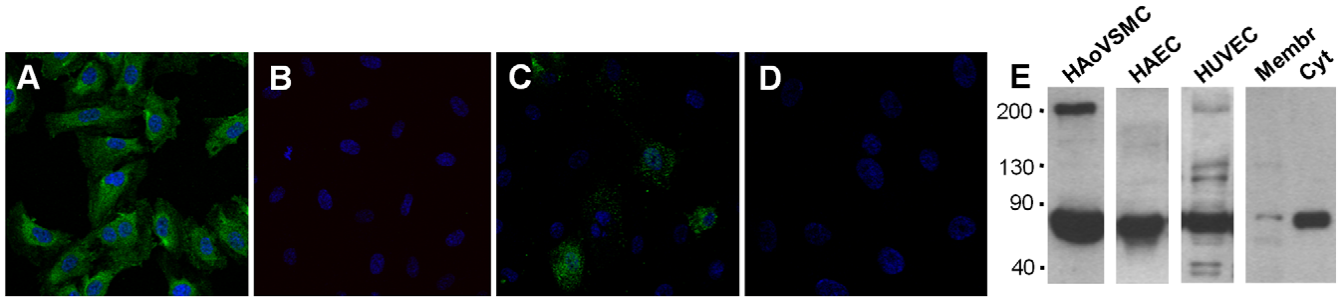
CaSR protein expression in HUVECs by Immunofluorescence Confocal Microscopy and Western Blot. Immunofluorescent localization of CaSR in HUVECs with specific antibody and negative control after fixation and permeabilization protocol (A and B), or after fixation but not membrane permeabilization (C and D). Representative Western Blot of CaSR protein levels in HAoVSMC, HAEC and HUVEC total lysate, and in HUVEC membrane and cytoplasm extracts (E).

To determine whether CaSR protein was expressed in HUVECs, immunofluorescence confocal microscopy analysis was performed using a monoclonal antibody against human CaSR. Excited ALEXA-488 emits green fluorescence. Nuclei stained with TO-PRO-3 Iodide display blue fluorescence. As shown in Fig. 1A, diffuse strong green fluorescence was observed in permeabilized HUVECs, demonstrating a mainly intracellular localization of CaSR protein. This evidence was confirmed by the low number of green fluorescent non-permeabilized HUVECs (Fig. 1C), thus demonstrating the major localization of the CaSR in the cytosol in this cell type despite the alleged plasma membrane localization of the mature functional protein. Again shown in Figs 1B and 1D, the absence of primary antibody completely abolished CaSR immunofluorescence, respectively in permealized and non-permeabilized HUVECs.

In order to better identify CaSR expression and cellular distribution, Western Blots were also performed. Fig. 1E shows the immunodetection of CaSR under reducing conditions. As compared to positive controls (Human Aortic Vascular Smooth Muscle Cells [HAoVSMCs] and Human Aortic Endothelial Cells [HAECs]) we identified not only a band of the size expected for the full-length CaSR monomer (100–130 kDa), but the appearance of strongly immunoreactive polypeptides of approximately 55–70 kDa and the presence of small polypeptides (30–40 kDa) which have been attributed to degradation of CaSR. Moreover, in agreement with our own observations on HUVECs, it has been reported that immunodetection of the CaSR isolated from HAoVSMCs shows a CaSR-specific immunoreactive band of approximately 200 kDa [26]. Thus, the observed pattern of bands (200, 100–130, 55–70 and 30–40 kDa) is consistent with results from others who have detected CaSR at several different molecular weights depending upon the extent of glycosylation of the proteins and whether the CaSR is seen as a monomer or multimer.

We also performed subcellular fractionation of the HUVECs to ascertain whether CaSR is located in the plasma membrane, in which case it would exert its currently known physiological function. Although we were consistently able to detect the widely described immunoreactive 55–70 kDa polypeptide in both subcellular compartments analyzed, it became evident from the Western Blot analysis that the subfraction containing plasma membranes was poorly enriched in an immunoreactive band of distinctively higher molecular mass (100–130 kDa, totally absent in cytoplasm fraction), suggesting the absence of the CaSR mature monomers, physiologically relevant in HUVECs. Since it has been reported [27] that the mature functional CaSR that resides on the cell surface is mainly in the form of a dimer corresponding to a molecular mass of approximately 250–280 kDa (or higher, depending on the extent and type of glycosylation), we also evaluated crude HUVEC protein extract. This molecular mass is not consistent with the band that we find (data not shown), suggesting the absence of the mature, physiologically relevant CaSR in HUVECs.

### Effects of R-568 and S-568 on HUVEC intracellular [Ca^2+^]_i_

To determine whether the addition of R- and S-568 might modulate [Ca^2+^]i in our cellular model, FURA-2AM-loaded HUVECs were stimulated using different concentrations (1–100 µM) of R-568 or its enantiomer S-568. The evaluation of [Ca^2+^]i was also performed in the presence or absence of Calhex-231 [28] at the highest calcimimetic concentration used (100 µM).

**Figure 2:**
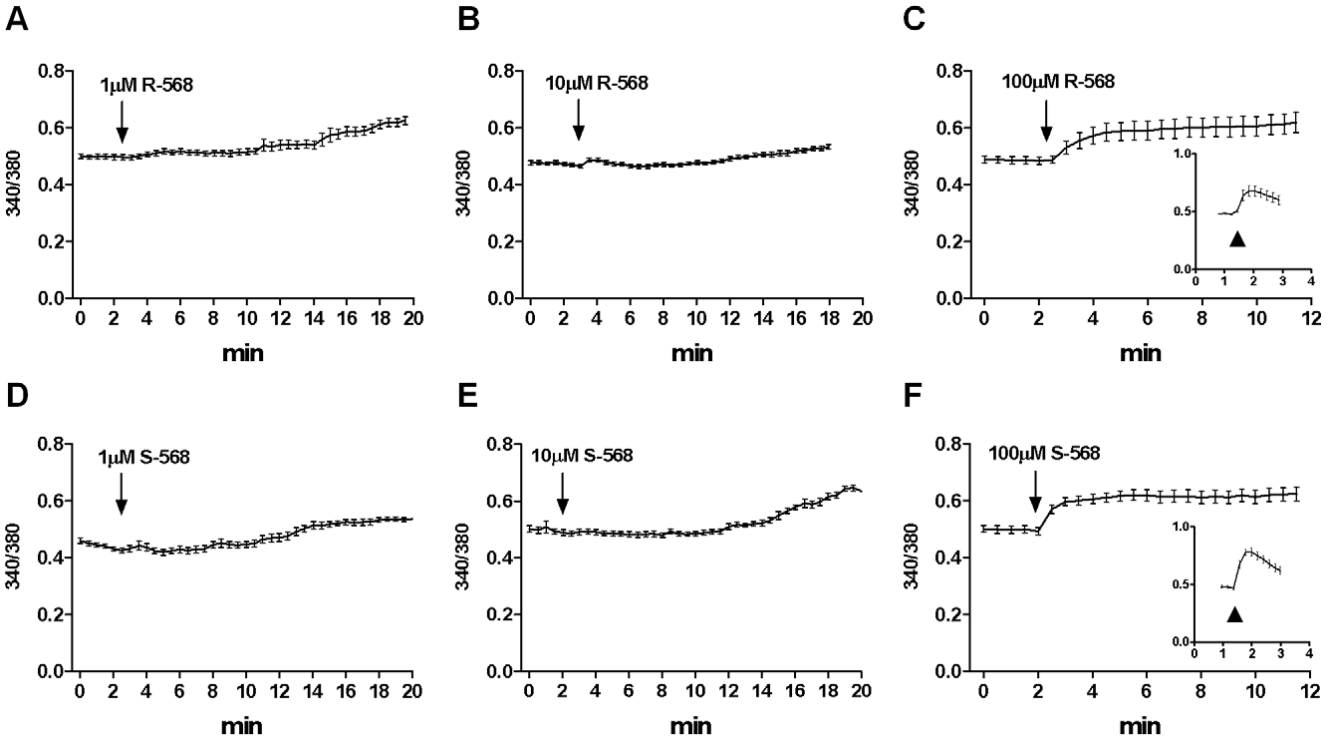
Effects of different doses of R-568 or S-568 on HUVECs [Ca^2+^]_i_. Traces (mean value ± SE) representing [Ca^2+^]i variations in FURA-2AM-loaded HUVECs stimulated with R-568 (A–C) or S-568 (D–F) at 1 µM (A and D), 10 µM (B and E) or 100 µM (C and F) concentrations. Representative traces after stimulation with R-568 100 µM + Calhex 10 µM (inset C) or S-568 100 µM + Calhex 10 µM (inset F).

As shown in Fig.2 (A, B, C), R-568 caused a dose-dependent increase in [Ca^2+^]i. in almost all stimulated cells (responsive cells were 92–100% of the cell population). At concentrations of 1 and 10 µM, R-568 induced a slow and delayed response, while at 100 µM, the compound produced a sustained rapid response. The enantiomer, S-568, had the same qualitative and quantitative effects as R-568 (Fig. 2, D, E, F). Notably, in the presence of the inhibitor Calhex 231, 100 µM calcimimetics significantly increased [Ca^2+^]i, thus demonstrating that CaSR is not involved in the action of R- and S-568 on the [Ca^2+^]i rise (inset Panels C and F).

### Role of [Ca^2+^]_o_ in R-568 and S-568 induced changes [Ca^2+^]_i_ in HUVECs

**Figure 3:**
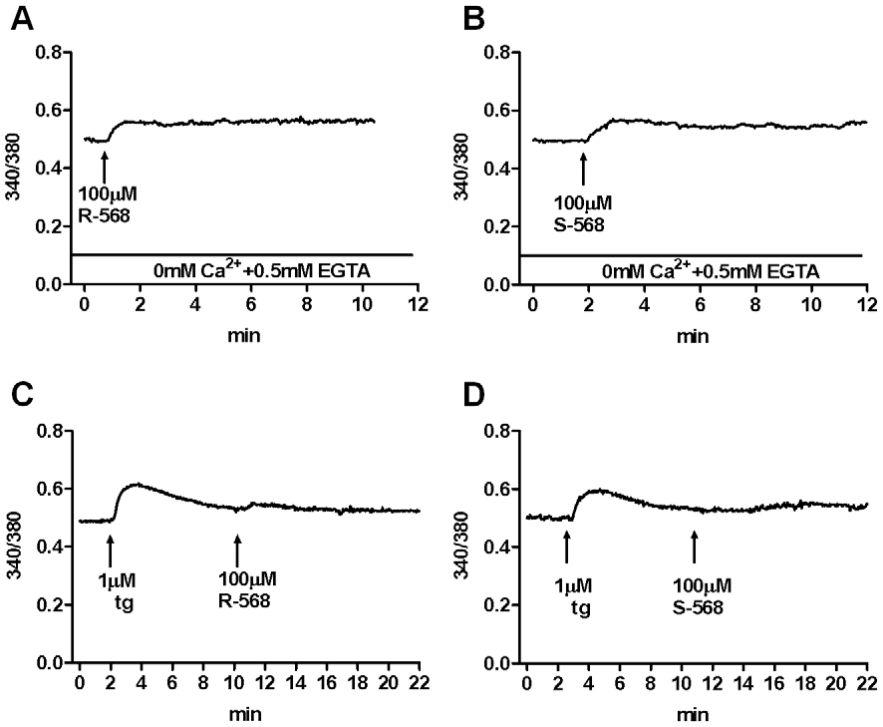
Effects of R-568 or S-568 on HUVECs [Ca^2+^]_i_ without extra or intracellular Ca^2+^. Representative Ca^2+^-variations in FURA-2AM-loaded HUVECs stimulated with 100 µM R-568 or 100 µM S-568 in Ca^2+^ free medium (A and B) or after depletion of intracellular Ca^2+^ stores by thapsigargin (tg) (C and D).

We tested whether in our cellular model the S- or R-568 triggered [Ca^2+^]i. rise was driven only by extracellular Ca^2+^ or also by Ca^2+^ release from internal stores. To this end, we performed experiments in an extracellular Ca^2+^-free condition (0mM Ca^2+^ + 0.5mM EGTA) and/or in an empty store condition, using thapsigargin (tg) to inhibit Ca^2+^ re-uptake by intracellular stores and consequently inducing store depletion [29]. In extracellular Ca^2+^-free conditions, R-568 and S-568 produced the same qualitative and quantitative effects as shown in complete medium (2mM Ca^2+^) (Fig. 3A and 3B). On the other hand, the presence of 1 µM tg inhibited the effect triggered by R-568 and/or S-568 (Fig. 3C and 3D), thus supporting the hypothesis that the R-568- or S-568-induced [Ca^2+^]i rise was due to ion release from intracellular stores.

In addition, when experiments were performed with the two conditions at the same time, the response to calcimimetic agents disappeared (data not shown).

### Effects of R-568 and S-568 on HUVEC NO production

**Figure 4:**
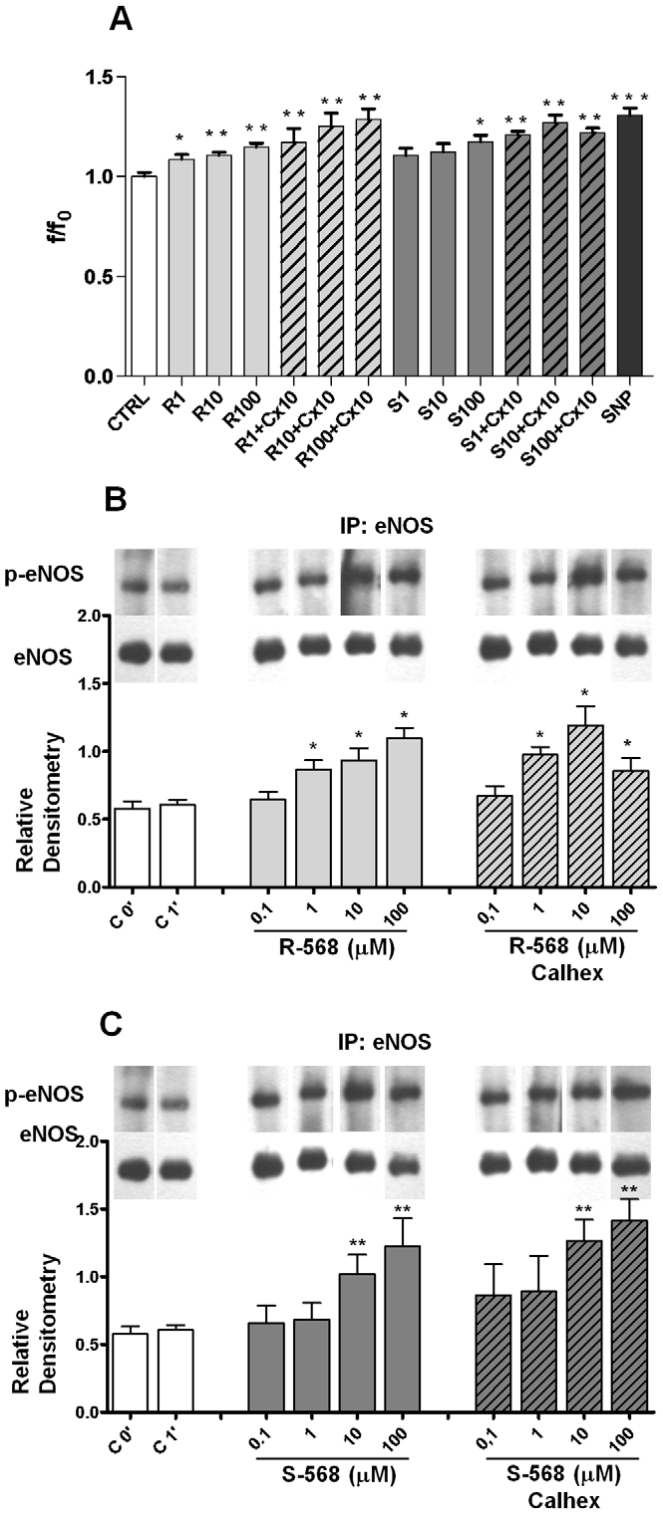
Effects of R-568 or S-568 +/- Calhex (10 µM) on HUVECs NO production. NO levels (mean value ± SE) in DAF-2DA-loaded HUVEC populations treated with R-568 or S-568 (1–100 µM) +/- Calhex 10 µM (A). eNOS-ser1177 phosphorylation in HUVECs stimulated with R-568 (B) or S-568 (C) (0.1–100 µM) +/- Calhex (10 µM). Representative immunoblot of eNOS-ser1177 phosphorylation (Upper Panel, B and C). Representative data from three experiments (means ± SD, Lower Panel, B and C). Phospho-eNOS (p-eNOS) expression was normalized vs total eNOS expression.

The effects of R-568 and S-568 on NO release from cultured HUVECs are shown in Fig. 4. In these experiments NO release was measured in the total cell population by a semi-quantitative and high-sensitive method, demonstrating that DAF-2DA-loaded cells were slightly but significantly stimulated by both R-568 (at doses of 1–100 µM) and S-568 (100 µM), in the presence of 2mM [Ca^2+^]o (Fig. 4A). The experiments were performed in the presence or absence of Calhex-231, a negative modulator of CaSR. In the presence of Calhex-231 (10 µM), R-568 stimulated NO release at all concentrations used. Similar data were obtained when S-568 was examined over the same concentration range, showing that there is no stereoselectivity in the responses. Thus, CaSR is unlikely to be involved in the action of R- and S-568.

In order to determine in our cellular model whether R-568 and S-568 stimulated NO release by modulation of ser1177 phosphorylation levels of eNOS, we evaluated the effect of calcimimetics on eNOS-ser1177 phosphorylation (p-eNOS) levels in the presence of 2mM [Ca^2+^]o. p-eNOS levels were investigated by Western Blot analysis using antibodies specific for the residue ser1177, which is phosphorylated in activated eNOS.

As compared to controls, R-568 and S-568 significantly stimulated eNOS-ser1177 phosphorylation in a dose-dependent manner, as assessed by evaluation of total and p-eNOS (Fig. 4B and 4C). Note that, parallel to calcimimetic stimulated NO production, eNOS-ser1177 phosphorylation levels were enhanced after R- and S-568 stimulation to a similar extent, regardless of whether they had been treated with Calhex-231. Thus, CaSR is not likely to be involved in this action by R- and S-568.

### Effects of extra- and intra-cellular calcium levels in R-568- and S-568-increased HUVEC NO release

**Figure 5:**
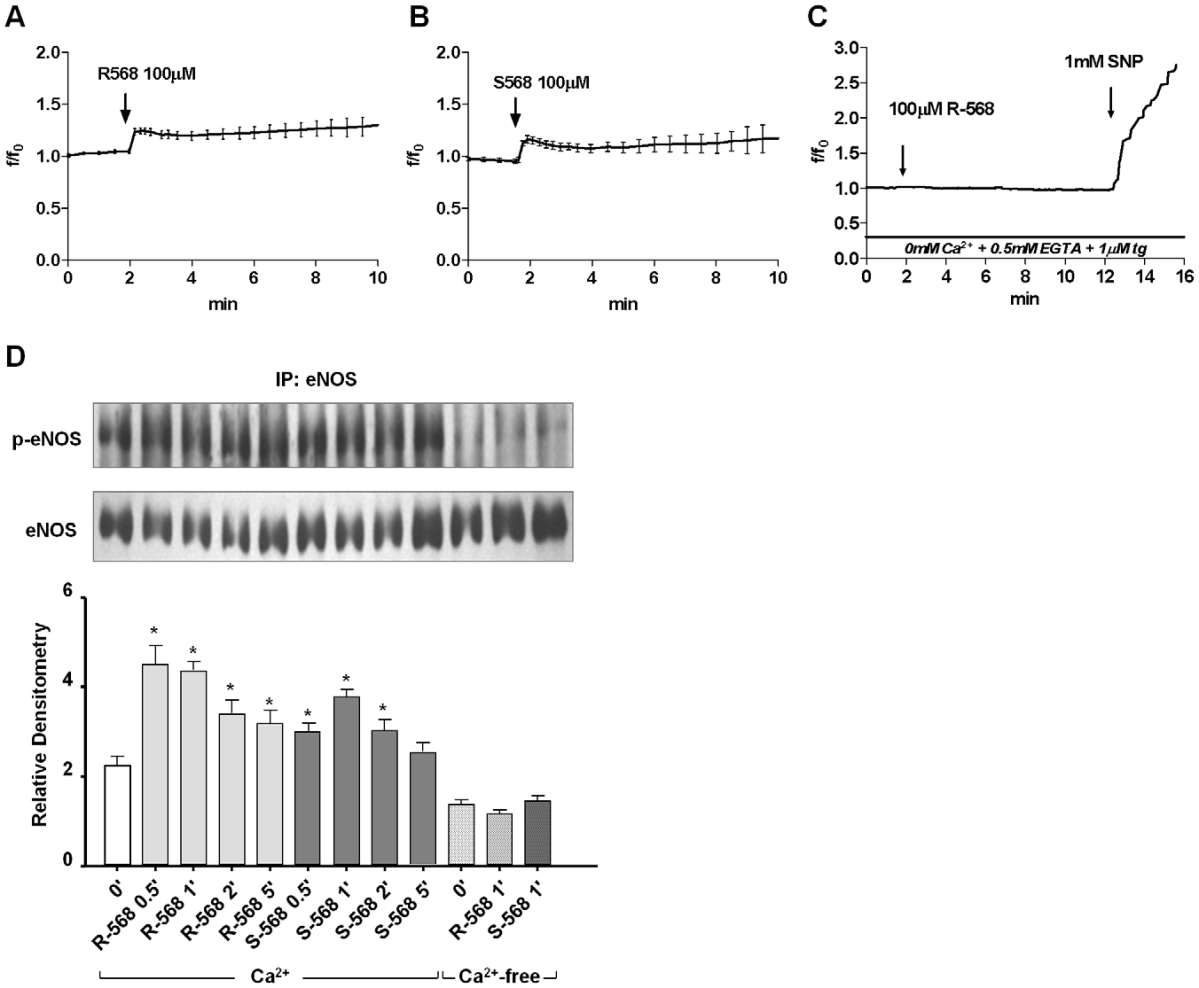
Effects of 100 µM R-568 and S-568 with and without extra and intracellular Ca^2+^ on NO production. Intracellular NO levels (mean value ± SE) in DAF-2DA-loaded HUVECs (A–C). Time effect of R-568 or S-568 100 µM on eNOS-ser1177 phosphorylation (D). Representative immunoblot of eNOS-ser1177 phosphorylation (Upper Panel). Representative data from three experiments (means ± SD, Lower Panel, p<0.003 vs 0′). Phospho-eNOS (p-eNOS) was normalized vs eNOS total.

Since a 100 µM concentration of either of R-568 or S-568 brought a sustained NO rise without any effect on cell morphology and adhesion features, we used this concentration to evaluate the role of calcium in calcimimetic-induced intracellular NO production in HUVECs by a single cell approach [30]. As expected, in the presence of 2mM [Ca^2+^]o, both 100 µM R- and S-568 brought a rapid and sustained intracellular NO rise in almost all cultured HUVECs (Fig. 5A, 5B). These effects appeared to be Ca^2+^-dependent, since R-568- or S-568-induced NO production was completely abolished in Ca^2+^-free conditions (Fig. 5C). In addition, when a NO donor (sodium nitroprusside, SNP) was added in these conditions, the gas was rapidly detected, thereby indicating the integrity of the experimental system (Fig. 5C).

In order to determine the dependence of R-568- and S-568-stimulated eNOS-ser1177 phosphorylation levels on modulation of [Ca^2+^]i, we also evaluated the effect of calcimimetics on p-eNOS levels in Ca^2+^-free conditions.

As compared to controls (time 0), R-568 and S-568 significantly (p<0.003) stimulated eNOS-ser1177 phosphorylation, which peaked at 0.5 to 1 minute of treatment, as assessed by evaluation of total and Ser1177-phosphorylated forms of eNOS (Fig. 5D). After 1 minute of calcimimetic incubation, their stimulatory effect on eNOS-ser1177 phosphorylation was totally abolished by Ca^2+^-free conditions, thus demonstrating that the mechanism leading to calcimimetic-induced NO release may potentially be mediated by both R- and S-568-increased intracellular [Ca^2+^]i levels and eNOS-ser1177 phosphorylation. In fact, it is known that agonist-stimulated intracellular calcium transients may form some of the initial steps in eNOS activation, and may be required for further activation of eNOS by other agonist-mediated pathways like phosphorylation.

### Effects of R-568 and S-568 on HUVEC and HAEC NOS activity

In order to confirm the calcimimetic role on NO release in HUVECs and to evaluate for the first time the potential effects of R- and S-568 on NO enzymatic release in HAECs we evaluated NOS activity by a highly sensitive method as the 3H-Arginine into 3H-Citrulline conversion assay.

As shown in Table 1, both cell strains were significantly stimulated by R-568 and S-568 at all doses employed (p<0.05). Preincubation with L-NAME (1 mM), a known NOS inhibitor, totally abolished the R- and S-568 increased NOS activity in both cell strains (data not shown). On the contrary, Calhex-231 (10 µM) did not significantly inhibit R- and S-568 stimulation of NO release at any concentration, confirming that there is no stereoselectivity in the responses.

**Table 1:** Effects of R-568 and S-568 on HUVEC and HAEC NOS activity.

	*HUVECs (pmoles NO/min/mg total protein)*	*HAECs (pmoles NO/min/mg total protein)*
*Control*	*0.15*±*0.01*	*0.12*±*0.01*
*R-568 (1* µ*M)*	*0.26*±*0.03* *	*0.24*±*0.02* *
*R-568 (10* µ*M)*	*0.28*±*0.02* *	*0.26*±*0.03* *
*R-568 (100* µ*M)*	*0.30*±*0.03* *	*0.23*±*0.01* *
*S-568 (1* µ*M)*	*0.24*±*0.01* *	*0.20*±*0.02* *
*S-568 (10* µ*M)*	*0.26*±*0.02* *	*0.22*±*0.03* *
*S-568 (100* µ*M)*	*0.27*±*0.03* *	*0.26*±*0.003* *

*p<0.05 (R- and S-568 vs Control).

## Discussion

The present study shows for the first time that, although CaSR protein was expressed in HUVECs, it was mainly distributed in cytoplasm while the functional CaSR dimers, usually localized on the plasma membrane, were absent. Nonetheless, both calcimimetics R- and S-568 significantly increased intracellular Ca^2+^ levels by mobilization of Ca^2+^ from intracellular stores, which in turn augmented NO release by a time- and Ca^2+^-dependent increase in eNOS-ser1177 phosphorylation levels. One should note that, in the presence of Calhex-231 (a negative modulator of CaSR), R-568 increased both [Ca^2+^]i levels and NO release at all concentrations used. Similar data were obtained when S-568 was examined over the same concentration range, showing that there is no stereoselectivity in the responses. Thus, CaSR is unlikely to be involved in the action of R- and S-568.

Our interest in this study stemmed from learning that, in addition to the known role of calcimimetics in regulating levels of PTH [1], a novel role has been proposed for calcimimetics as vasculotropes [31]. This is supported by the demonstration that CaSR is expressed in the arterial wall [26], both in vascular arterial endothelial and in smooth muscle cells [10], [11], [32], as well as by animal model studies showing a direct significant beneficial effect by R-568 on hypertension and on calcification and vascular remodelling in both uremic and control rats [17]. No studies in humans have been designed so far to evaluate any direct cardiovascular effects by calcimimetics. However, a recent prospective study did show that after a 6-month follow-up, the mean blood pressure in 14 renal transplant recipients receiving cinacalcet for persistent hyperparathyroidism diminished significantly (from 94.1 to 88.0 mmHg, p<0.019) with no changes in antihypertensive treatment [33]. Moreover, data obtained in over 600 End-Stage Renal Disease (ESRD) patients from phase 3 studies with the calcimimetic agent cinacalcet indicate lower systolic and diastolic blood pressure (BP) values after one year of treatment (144.4±2.4 and 78.6±1.5 versus 138.6±2.4 and 76.9±1.5 mmHg, respectively) (personal communication, U. Fraass, Amgen, March 2005). Clearly, since it is not yet clear whether the in vivo vascular function of R-568 is related to its effect on systemic calcium homeostasis or exerts a direct effect on the vasculature, additional focused studies are needed to establish the impact of calcimimetic agents on potential NO release and BP regulation in ESRD, a pathological state characterized by reduced vascular NO bioavailability [34].

On the other hand, experimental studies ex vivo as well as on cultured cells support the hypothesis that both R- and S-568 may play a direct role in vascular functions. For example, in in vitro and ex vivo experiments, activation of CaSR seems to have a potential role in the control of vasodilation regulating the myogenic tone in rat subcutaneous arteries, while treatment with the type II CaSR agonist cinacalcet results in a concentration-dependent vasodilatation of isolated precontracted aortae [12].

Although these data suggest a direct effect by calcimimetics on the vasculature and in particular on the modulation of NO bioavailability, which is known to play a key role in the regulation of endothelial-mediated vasodilation and vascular homeostasis [21], at present there are no in vitro studies demonstrating the cellular mechanism(s) potentially involved in the in vivo vascular effects observed with R-568.

Based on recent in vitro evidence from cultured human aortic endothelial cells showing that these cells express CaSR and that the agonist spermine induces an increase in [Ca^2+^]i leading to the production of NO [11], our study demonstrates that both R-568 and its enantiomer S-568 are able to increase NO release both in HAECs and in HUVECs through eNOS activation. Although these calcimimetic concentrations (1–100 µM) are 10–100 times higher than those needed to treat uremic hyperparathyroidism in vivo [6], [7], they are close to the levels that the drugs reach in animal models after oral administration. In support of this, Nakagawa et al. [23] demonstrated that in vivo both R-568 and S-568 increased mesenteric and renal blood flow when organ artery blood concentrations of both compounds reached 70 µM, leading us to hypothesize that the in vivo vascular effects of these compounds may be mediated by increased endothelial NO release. Notably, the effects of S-568 in these experiments were almost identical with those of R-568, indicating a CaSR-independent mode of action.

In agreement with these findings, our in vitro data show that in HUVECs both R- and S-568 exert the same effects and in particular their ability to increase [Ca^2+^]i (Figs 2 and 3), NO levels (Figs 4 and 5) and NOS activity in HUVECs and HAECs (Table 1) is unaffected by Calhex-231 (a negative modulator of CaSR), supporting the idea of a potential vascular CaSR-independent rather than CaSR-dependent effect.

Regarding the mechanism(s) potentially involved in explaining the vasculotropic effect of such calcimimetics, our data show that the proven ability of calcimimetics is unaffected in Ca^2+^-free medium conditions while it is abolished after depletion of the intracellular Ca^2+^ pool by thapsigargin, indicating that the effect is independent of [Ca^2+^]o and possibly mediated by no stereoselective R-568 and S-568 interaction with G protein-coupled receptors. Note that activation of this kind of receptor is known to trigger complex intracellular signals through G-protein and phospholipase-C, which in turn stimulate inositol-triphosphate production and thereby increase intracellular Ca^2+^ release [5], [11].

In support of this hypothesis, recently GPRC6A, a novel G protein-coupled receptor (designated family C, group 6, subtype A) that is sensitive to Ca^2+^ and closely related to CaSR [13], has been identified in endothelium of rat mesenteric and coronary arteries [14] and, notably, can be activated by NPS R-568 [15], a known positive allosteric modulator of CaSR.

Thus, we may conjecture that in HUVECs R-568 or S-568 might allosterically modulate a member of the GPCR family C or specifically GPRC6A activity and in turn activate various intracellular pathways leading to increased eNOS activity and then NO production.

Interestingly, in our cellular model the [Ca^2+^]i increase drives both R- and S-568 NO production. Thus, when [Ca^2+^]i increase is almost completely prevented, the R- and S-568 effects on NO release are totally abolished (Fig. 5). The mechanism may be clarified by reflecting that in endothelium NO release is due to eNOS activation which results from a complex combination of protein-protein interaction and signal transduction cascades involving calcium mobilization and/or phosphorylation events. In particular, several agonists that raise intracellular calcium concentrations, such us bradykinin, promote Ca^2+^/calmodulin binding to eNOS and caveolin dissociation from the enzyme, resulting in an activated eNOS-complex. This concept has been greatly refined to include a complex array of protein-protein interactions that can lead to increased eNOS phosphorylation due to interaction with Hsp90. In fact, the interaction between eNOS and Hsp90 can induce formation of ternary complex which includes the kinase Akt. This might significantly contribute to increasing p-eNOS levels and then eNOS activity [35].

Moreover, multiple protein kinases can modify eNOS activity through effects on serine phosphorylation at position 1177, which, in turn, influence NO release [36].

In point of fact, in our experimental model both R- and S-568 rapidly and significantly increased eNOS-ser1177 phosphorylation levels (p<0.003) and these calcimimetic effects on eNOS phosphorylation disappeared in Ca^2+^-free conditions, indicating that both R- and S-568 stimulated NO release needs [Ca^2+^]i in order to rise (Fig. 5).

From these data it is conceivable that the potential non stereospecific activation of a G protein-coupled receptor by R-or S-568 elicits additional intracellular signals, potentially through interaction with Gαq/11 subunit heterotrimeric G-proteins, resulting in potential activation of a PI3-Kinase/Akt cascade leading to increased eNOS phosphorylation levels [5].

Moreover, since the activation of eNOS in endothelial cells involves a complex set of molecular events all acting in concert to increase eNOS activity, further studies will be needed to evaluate the potential role of R-568 or S-568 in the modulation of eNOS activity via intracellular enzyme compartmentalization and/or protein-protein interactions [36].

The present study provides further insight into the potential CaSR-independent novel role of calcimimetics in vascular biology, by showing that these compounds increase NO release in cultured HUVECs. It should be noted, however, that, as with the CaSR-independent vasodilatory action of both R-568 and S-568 demonstrated by Nakagawa et al. [23], so the in vitro effect was observed with high compound concentrations, exceeding those required for modulation of PTH secretion. Thus, the implications of such vasculotropic effects for clinical purposes remain to be established.

Nevertheless, that [Ca^2+^]o-independent endothelial NO production was increased by calcimimetics, as observed in our study, may support the previous suggestion by Al-Aly [31], namely that CaSR and/or other mechanisms may be implicated in several cellular processes and that translational applications of calcimimetics may not be exclusively calcium-centric. This provides a new impetus for further investigations aiming to characterize calcimimetics as vasculotropes, which would open new perspectives as to the CaSR-independent role of these compounds in regulating the endothelial function in uremia and, more generally, as to their role as vasculotrope agents.

## Materials and Methods

### Chemicals

Powered R-568-HCl and S-568-HCl were provided by Amgen (Amgen, Inc., Thousand Oaks, CA, USA), resuspended in water at 2 mM concentration and stored at −20°C. Calhex-231 (Santa Cruz, sc-207394) from Santa Cruz was resuspended in Ethanol at 10 mM concentration and stored at −20°C.

### Ethics Statement

Umbilical cords were obtained from randomly selected healthy mothers delivering at the Chieti and Pescara University Hospital. All procedures were in agreement with the ethical standards of the Institutional Committee on Human Experimentation (“University G. d'Annunzio Ethics Committee review board”, Reference Number: 1879/09COET) and with the Declaration of Helsinki Principles. After approval of the protocol by the Institutional Review Board, signed informed consent form was obtained from each participating subject.

### Cell Culture

Primary HUVECs were obtained and cultured as previously described [37]. Briefly, after perfusion of umbilical cords with 0.1% collagenase at 37°C, HUVECs were grown on 0.2% gelatin-coated tissue culture plates in 50∶50 Dulbecco's Modified Eagle's Medium-Low Glucose (DMEM) (PAA E15-806) and M199 (PAA E15-834), supplemented with 20% FBS (PAA A15-101), 10 µg/ml heparin, and 50 µg/ml ECGF (Sigma, Saint Louis, USA), 100 U/ml penicillin-100 µg/ml streptomycin (PAA P11-010), 2 mM L-Glutamine (PAA M11-004). In all experiments, cells were used between the third and sixth passages in vitro and starved for 2 hours in EBM (Endothelial Basal Medium, without Fenol Red; Lonza Clonetics CC-3129) supplemented with 1% platelet-deprived horse serum and 100 µM L-arginine. HAoVSMCs and HAECs (ATCC, USA) were employed as positive controls. HAECs were also employed to evaluate NOS activity by measuring the conversion of L-[^3^H]-arginine into L-[^3^H]-citrulline.

### Immunofluorescence

For Immunofluorescence Confocal Microscopy experiments, HUVECs were plated onto glass gelatin-coated coverslips in 6-well plates and grown at sub-confluence. Briefly, cells were fixed for 10 min at room temperature with a 3% paraformaldehyde solution in 1X-Dulbecco's phosphate buffered saline (PBS) 2% sucrose (fixation solution pH 7.6). Then, cell membranes were permeabilized (0.5% Triton X-100 20mM HEPES, 300mM sucrose, 50mM NaCl, 3mM MgCl_2_, pH 7.6) for 5 min at room temperature. Some not permeabilized samples were left in PBS for 5 min at room temperature. For CaSR/nucleus immune-labeling, cells were blocked with 10% Bovine Serum Albumine (BSA) in PBS for 30 min at room temperature, followed by 60 min incubation at 37°C in a 1∶100 dilution in 1% BSA in PBS of anti human CaSR Mouse monoclonal antibody (MA1-934 ABR Affinity BioReagents, CO, USA) raised against a synthetic peptide corresponding to residues 214–235 of hCaR protein, at a final concentration of 10 µg/ml. Finally, Alexa-488 (Molecular Probes A11001) goat anti-mouse secondary antibody (1∶1000) in 1% BSA/PBS was incubated for 1 h at room temperature. For nuclear staining, TO-PRO-3 Iodide (Molecular Probes T3605) in a 1∶100 final dilution was added during the last 15 min of incubation. Negative controls were processed as for CaSR staining, but incubated only with Alexa-488 goat anti-mouse secondary antibody. All the slides were mounted with Slowfade (Molecular Probes), and observed with a ZEISS 510META Confocal Microscope. Images were acquired using LSM 510 META confocal microscopy software (rel. 3.0, ZEISS).

### Western Blot Analysis

Total protein content, membrane and cytoplasm protein fractions were isolated from untreated HUVECs and quantified using the BCA Protein Assay Kit (Pierce Biotechnology, Rockford, IL, USA). 30 µg of HUVECs, of HAoVSMCs and HAECs (positive controls) total protein lysates, and 15 µg of membrane and cytoplasm extracts were heated at 100°C for 10 min in an SDS sample buffer with 2-Mercaptoethanol (reducing conditions), electrophoretically separated by 4–10% SDS-PAGE under reducing conditions and electroblotted on nitrocellulose membrane. The membranes were blocked in 5% milk solution in TBS-Tween 20 0.1% (TBST) for 1 hour at room temperature and probed with a 1∶2000 dilution of anti-human CaSR mouse monoclonal antibody (MA1-934 ABR) and with a 1∶10000 dilution of anti-human β-Actin mouse monoclonal antibody (Sigma A5441) in 5% milk solution in TBS-Tween 20 0.1% at 4°C overnight. After washing, the membranes were further incubated with a 1∶10000 dilution of Goat Anti-Mouse IgG specific Peroxidase conjugated (Calbiochem, Merck, 401253) in 5% milk solution in TBS-Tween 20 0.1% for 90 min at room temperature. Immunoreactive bands were detected using the ECL system (Amersham, GE Healthcare).

### [Ca^2+^]_I_ measurement

For [Ca^2+^]i measurements HUVECs (6000 cells/well) were plated on black 96-well plates with a clear bottom in complete medium. After 1 day the cultures were serum-starved for 2 hours in EBM (phenol red-free endothelial basal medium) supplemented with 1% platelet-deprived horse serum and 100 µM L-arginine. In the last 45 min of serum-starvation 5 µM FURA-2AM was added to the cells, then rinsed with Standard Medium (SM; 125mM NaCl, 5mM KCl, 1mM MgSO_4_, 1mM KH_2_PO_4_, 5.5mM Glucose, 2mM CaCl_2_, 20mM HEPES, pH 7.4) and bathed in Ca^2+^-containing SM. FURA-2AM-loaded cells were sequentially excited at 340 and 380nm by a high-speed wavelength switcher (PolychromeII, Till Photonics, Germany) equipped with 75W-Xenon lamp (Ushio Inc., Japan) and stimulated with R-568 or S-568 (1–10–100 µM). The effect of Calhex was tested on R-568 or S-568 100 µM. To study the role of extra- and intra-cellular Ca^2+^,the cells were stimulated with R-568 or S-568 100 µM respectively in Ca^2+^-free SM and after depletion of intracellular Ca^2+^ stores by thapsigargin. The fluorescence images were acquired every 2 sec by a C6790 cooled CCD camera (Hamamatsu Photonics, Hamamatsu, Japan). The image ratio calculations were carried out on a pair of corresponding 340 and 380 image files. The graphs show the temporal plots calculated from the image ratios (340/380). For each experimental condition, 10 different wells were observed to analyze at least 35–50 cells).

### Measurement of intracellular NO levels

The total NO production was measured in HUVEC populations using a modified version of the method by Wang and Joseph [38]. NO production was studied by using DAF-2DA (Calbiochem, Merck 251505) in the same culture conditions as used for evaluating [Ca^2+^]i variation. In the last 30 minutes of serum-starvation 10 µM DAF-2DA was added to the cells, which were finally rinsed in Standard Medium (SM; 125mM NaCl, 5mM KCl, 1mM MgSO_4_, 1mM KH_2_PO_4_, 5.5mM Glucose, 2mM CaCl_2_, 20mM HEPES, pH 7.4). DAF-2DA-loaded cells were stimulated with R-568 or S-568 (1–10–100 µM) with or without Calhex 231 (10 µM) in Ca^2+^-containing SM or Ca^2+^-free SM plus thapsigargin. The NO donor Sodium Nitroprusside (SNP) was used as a internal control. The fluorescence, read by a microplate-reader (SPECTRAmax GEMINI XS, Molecular Devices, Toronto, Canada) at 490nm excitation/510nm emission, was acquired from each sample (n = 8) and expressed as a mean ± SE of the f/fc ratio (f = stimulated cells; fc = control cells).

NO production was studied in detail in the same culture conditions using the method previously reported by Di Pietro et al., 2006 [30]. Briefly, DAF-2DA-loaded cells bathed in either Ca^2+^-containing SM or in Ca^2+^-free SM (CaCl_2_ was substituted by 2mM MgCl_2_ plus 0.5mM EGTA) plus 1 µM thapsigargin administration, were excited at 490nm, and during fluorescence recording were stimulated with R-568 or S-568 (1–10–100 µM). The fluorescence images were sampled every 2 sec and traces were calculated as f/f0 (f = fluorescence emission of a single DAF-2DA-loaded cell; f0 = fluorescence value of the same cell before the addition of stimuli). At the end of each experiment, the NO donor SNP (sodium nitroprusside) was used as an internal control. For each experimental condition 10 different wells were observed so as to analyze at least 35–50 cells).

### eNOS-ser1177 phosphorylation analysis

HUVECs were grown at sub-confluence on Petri dishes 100mm in complete medium and then serum-starved as described above. To study the effects of different doses of R-568 or S-568 (0.1–1–10–100 µM ) +/- Calhex (10 µM) on HUVECs NO production, the cells were bathed in Ca^2+^-containing SM and stimulated for 1 minute. To study the effects of R-568 and S-568 (100 µM ) with and without extra- and intracellular Ca^2+^ conditions on NO production, the cells were treated with R-568 or S-568 (100 µM) time dependently (0, 0.5, 1, 2, 5 minutes) in Ca^2+^-containing SM and for 1 minute in Ca^2+^-free SM. Total protein lisates were recovered and quantified. eNOS-ser1177 phosphorylation levels were determined by Western Blot after immunoprecipitation. Briefly, 500 µg of lysate proteins were incubated overnight with 5 µg of eNOS mouse monoclonal antibody (Clone3, 610297 BD Transduction labs) at 4°C, gently rocking. The next day 50 µl of 50% slurry protein A/G beads (sc-2003 Santa Cruz) was added to the mixture and incubated for 4 hours at 4°C, gently rocking. After 3 washes with IP buffer (50nM HEPES pH 7.2, 1nM MgCl_2_, 1nM CaCl_2_, 2nM NaVO_3_, 10nM Na Pyrophosphate, 10nM NaF, 2nM EDTA pH 8, 10% glycerol, 1% NP40, 137nM NaCl, 200nM PMSF, 1 µg/ml aprotinin, 1 µg/ml leupeptin and 1 µg/ml peptastatin) immunoprecipitates were resolved on an 8% SDS-PAGE gel and transferred to nitrocellulose. Then, membranes were blocked for 1 hour in 5% BSA in TBST and incubated with a 1∶1500 dilution in 1% BSA of eNOS-Phospho ser1177 rabbit polyclonal primary antibody (07–428, Upstate Biotechnology) overnight at 4°C. After three washes with TBS-Tween 20 0.1%, membranes were incubated with a 1∶2000 dilution in 5% non-fat milk in TBST of anti-rabbit IgG-HRP conjugate secondary antibody (Santa Cruz sc-2004) for 90 minutes at RT. Immunoreactive bands for eNOS-Phospho ser1177 were visualized by ECL Plus detection reagent (Amersham Pharmacia). To determine eNOS total content the membranes were stripped using a Restore Blot Stripping Buffer (Thermo Scientific 21059) following the manufacturer's instructions and then re-probed with a 1∶2000 dilution in 5% non-fat milkof the eNOS mouse monoclonal antibody overnight at 4°C. After three washes with TBST, membranes were incubated with anti-mouse IgG-HRP conjugate secondary antibody (Calbiochem 401253, 1∶10000 in 5% non-fat milk) for 90 minutes at RT. Immune complexes were visualized by ECL and quantified by scanning densitometry (Kodak digital photocamera connected to a computer). The data were elaborated by the Bio-Rad Image Processing and Analysis System. Phospho eNOS was normalized vs eNOS total.

### NOS activity 

NOS activity was evaluated by measuring the conversion of L-[^3^H]-arginine into L-[^3^H]-citrulline as described by [39]. Briefly, HUVECs and HAECs were grown at confluence and were stimulated with calcimimetics (1–100 µM) and/or Calhex-231 (10 µM). After that, cells were detached and then resuspended in 0.2 ml of reaction buffer (20 mmol/L Hepes-Na^+^, 0.5 mmol/L EDTA, 1 mmol/L dithiothreitol, pH 7.2) and sonicated on ice. In each test tube the following reagents were added to 100 µl lysate at the final concentrations: 2 mmol/L NADPH, 1.5 mmol/L CaCl_2_, 0.1 mmol/L BH_4_ (tetrahydrobiopterin) (Sigma-Aldrich, MO, USA), 2.5 µCi L-[^3^H]-arginine ( = 0.4 µM) (Amersham Pharmacia Biotech, PA, USA). After 15 min incubation at 37°C, the reaction was stopped by adding 2 ml Hepes-Na^+^ pH 6 containing 2 mmol/L EDTA; the entire reaction mixture was applied to 2 ml columns of Dowex AGWX8-200 (Aldrich, Steinheim, Germany) (Na^+^ form) and eluted with 4 ml of water. The radioactivity corresponding to L-[^3^H]-citrulline content in the eluate was measured by a liquid scintillation analyzer (Packard Bio Science Company, Meriden, CT, USA). In some experiments, L-NAME (1mM/L; Sigma Chemicals) was added 40 min before adding L-[^3^H]arginine. NOS activity was expressed as pmoles citrulline/min/mg protein.

### Statistical analysis of data

Data are reported as means ± SE or SD. Statistical comparisons were made using Student's t test for paired and unpaired groups. An analysis of variance was used when multiple comparisons were performed. A difference was considered significant at p<0.05.
